# Recent Advances in Cyanine-Based Phototherapy Agents

**DOI:** 10.3389/fchem.2021.707876

**Published:** 2021-06-24

**Authors:** Kubra Bilici, Sultan Cetin, Eda Celikbas, Havva Yagci Acar, Safacan Kolemen

**Affiliations:** ^1^ Department of Chemistry, Koc University, Istanbul, Turkey; ^2^ Surface Science and Technology Center (KUYTAM), Koc University, Istanbul, Turkey; ^3^ Graduate School of Materials Science and Engineering, Koc University, Istanbul, Turkey; ^4^ Boron and Advanced Materials Application and Research Center, Koc University, Istanbul, Turkey; ^5^ TUPRAS Energy Center (KUTEM), Koc University, Istanbul, Turkey

**Keywords:** cancer, photothemal therapy, photodynamic therapy, cyanine, synergistic therapy

## Abstract

Phototherapies, in the form of photodynamic therapy (PDT) and photothermal therapy (PTT), are very promising treatment modalities for cancer since they provide locality and turn-on mechanism for toxicity, both of which are critical in reducing off-site toxicity. Irradiation of photosensitive agents demonstrated successful therapeutic outcomes; however, each approach has its limitations and needs to be improved for clinical success. The combination of PTT and PDT may work in a synergistic way to overcome the limitations of each method and indeed improve the treatment efficacy. The development of single photosensitive agents capable of inducing both PDT and PTT is, therefore, extremely advantageous and highly desired. Cyanine dyes are shown to have such potential, hence have been very popular in the recent years. Luminescence of cyanine dyes renders them as phototheranostic molecules, reporting the localization of the photosensitive agent prior to irradiation to induce phototoxicity, hence allowing image-guided phototherapy. In this review, we mainly focus on the cyanine dye–based phototherapy of different cancer cells, concentrating on the advancements achieved in the last ten years.

## Introduction

Photothermal therapy (PTT) and photodynamic therapy (PDT) are two different light-based therapeutic approaches for the treatment of various cancers (Jaque et al., 2014; Li et al., 2018b; Lan et al., 2019; Li X. et al., 2020; Wang et al., 2021). Generation of phototoxicity by the irradiation of photosensitive agents with no dark toxicity provides a long-desired locality in cancer treatment with an additional turn-on mechanism, which dramatically reduces the off-site toxicity. No cumulative toxicity, lack of resistance to multiple applications of phototherapies, and indications of reversal of multidrug resistance and influence on the metastatic tumor (Guo et al., 2017; Wei et al., 2019) accelerated the research efforts on phototherapy of cancer (Li et al., 2016; Jung et al., 2018; Dos Santos et al., 2019). PTT and PDT differ in the type of phototoxicity they generate. PTT is based on local temperature increase (Hu et al., 2018; Jung et al., 2018; Lv et al., 2020), and PDT is triggered by formation of reactive oxygen species, especially singlet oxygen (^1^O_2_) (Celli et al., 2010; Li X. et al., 2017; Li et al., 2018a) by irradiation of a photosensitizer (PS) at a molecule-specific wavelength.

A successful PS should fulfill the following requirements: 1) low dark toxicity, 2) strong absorbance at the excitation wavelength, 3) high ^1^O_2_ quantum yield for PDT and high photo to thermal conversion efficiency for PTT, and 4) tumor-targeting ability to induce tumor-selective, strong phototoxicity (Jaque et al., 2014; Hu et al., 2018; Lan et al., 2019). Besides, absorbance at long wavelengths and phototoxicity generated at low power densities for safety and penetration depth are also critical in the clinical translation of these approaches.

As all therapeutic methods, PDT and PTT have limitations that need to be circumvented for broader use and improved therapeutic outcomes. One strategy to achieve this is to combine PDT with PTT, which seems to provide a synergistic effect (Bharathiraja et al., 2018; Bilici et al., 2019; Wang et al., 2019; Xia et al., 2019). Practically, if this can be achieved with a single PS, clinical translation would be relatively easy. The portfolio for such PSs is minimal, but cyanine dyes demonstrate such potential, and hence are subject to extensive research. In this review, we have summarized the PDT, PTT, and synergistic PTT + PDT approaches utilizing cyanine derivatives.

## Photodynamic and Photothermal Therapy

PDT has three critical components that play a role in cell death: light, photosensitizer (PS), and molecular oxygen (^3^O_2_). A PS is a molecule that transfers the absorbed energy from the light irradiation to form reactive oxygen species (ROS) or singlet oxygen (Kwiatkowski et al., 2018; Dos Santos et al., 2019). When treated with light, the PS absorbs the light and is excited from the ground state to the excited singlet state. This excited PS is unstable and can either emit the excess energy as light/heat or undergo intersystem crossing (ISC), whereby the spin inverts and transforms into its triplet state, which is relatively more stable than the singlet state. There are two alternative relaxation pathways for the triplet PS. It can react with an organic molecule from its surroundings, such as the cell membrane, and pick up a hydrogen atom or an electron to form radicals. These radicals then interact with oxygen and form ROS [e.g., superoxide anion (O_2_^−^), hydrogen peroxide (H_2_O_2_), and hydroxyl radical (HO^•^)], which are the so-called type I reactions. In type II reactions, the triplet state PS can transfer its energy directly to the tissue oxygen and forms singlet ^1^O_2_. Both of these reactions take place simultaneously, and due to its simplicity, it is believed that most of the PS undergoes Type II reaction (Kwiatkowski et al., 2018). However, the contribution from both mechanisms depends on the level of hypoxia, pH, and the nature of the PS (Lucky et al., 2015). The effect of PDT on cancer cells can be summarized as follows: direct cytotoxic effect, damage to the vascular system, and stimulation of the immune system (Agostinis et al., 2011). As ROS has a very short half-life in aqueous solutions, only the cells in the PS range are affected by the PDT. This is advantageous to protect the surrounding healthy tissue but also limits the success of PDT when solid tumors are considered. It is worth underlining that the efficiency of the PDT highly depends on the PS type, tumor accumulation, administered dose, light exposure, oxygen availability, and singlet oxygen generation efficiency of the PS (Dolmans et al., 2003). Tumor hypoxia, quick clearance of PS, low efficiency, poor solubility or instability of the PS molecules, photobleaching, and day-light sensitization, plus the limited penetration depth of most commonly used red light, limit the clinical success of the PDT and require significant improvement (Deng et al., 2021).

In the case of PTT, irradiation of a PS causes local temperature increase by converting NIR light to heat, which may thermally ablate tumor cells or induce mild hyperthermia (Johannsen et al., 2005; Habash et al., 2007). Hyperthermia and thermal ablation treatments occur between 48 and 60°C temperature range, respectively (Habash et al., 2006; Habash et al., 2007). In hyperthermia, the local temperature increases to 41–48°C and cells should be kept at this temperature for about 15–60 min for the cell death; however, a few minutes at 48–60°C can kill the cells *via* thermal ablation (Habash et al., 2006). It is well established that thermal ablation triggers necrosis (Jaque et al., 2014). Although some organic molecules can be used as a PS for PTT (Lv et al., 2020), recently metal nanoparticles (Ke et al., 2011; He et al., 2014), metal oxide nanoparticles (Chu et al., 2013; Bilici et al., 2018), and quantum dots (Liang et al., 2018; Hashemkhani et al., 2020), especially with significant absorbance at near-infrared (NIR) for both better tissue penetration and safety due to its nonionizing nature (Jaque et al., 2014), are preferred. Light-to-heat conversion efficiency, dark toxicity, selective tumor accumulation, photostability, and effective heating of the whole tumor are factors to consider for PTT. Higher irradiation power requirement and tumor ablation causing necrosis of the healthy tissue on the path make low-temperature PTT more attractive but less effective if not assisted by a secondary treatment modality, preferentially PDT.

It has been reported that apoptotic cell death induced by PDT causes the release of signaling molecules to trigger immune cell death, which is quite important for complete treatment, treatment of metastatic tumor, and prevention of reoccurrence. On the other hand, PTT was reported to cause significant influence in suppression of drug resistance, increase in blood flow which would bring in oxygen to the hypoxic tumor, and improve permeability and hence improve penetration of PS into the tumor (Deng et al., 2021; Ding et al., 2021; Ming et al., 2021). All of the individual strengths of PTT and PDT point out not only a way to overcome their limitations but also a tremendous potential synergism to maximize the therapeutic effect.

## Cyanine Dyes in Phototherapies

Several small organic molecules appeared as phototherapy agents for the treatment of various cancer types (Luo et al., 2016; Tan et al., 2017; Wang et al., 2019). Cyanine derivatives, either indocyanine green (Yuan et al., 2013) or heptamethine cyanine (Shi et al., 2016), are the most preferred and promising ones since they induce both PDT and PTT. Additionally, some of the cyanine derivatives can be targeted to mitochondria due to their cationic lipophilic nature (Weinberg and Chandel, 2015; Shi et al., 2016; Jung et al., 2017; Zhou et al., 2019). In the recent years, decorating these dyes with different heavy atoms improved the efficacy of the synergistic phototherapy by addressing the low singlet oxygen generation capacity of the cyanine cores (Gorman et al., 2004; Atchison et al., 2017; Usama et al., 2018; Xu et al., 2019). In this review, we summarized PDT, PTT, and synergistic PTT + PDT approaches utilizing cyanine derivatives.

### Cyanines

**FIGURE 1:**
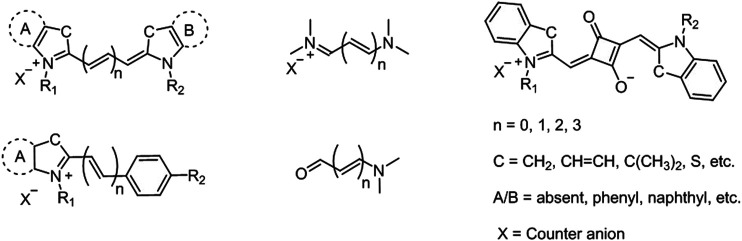
Representative structures of cyanine fluorophores.

Cyanine dyes are characterized by two nitrogen heterocyclic rings connected *via* a π-conjugated polymethine chain (Figure 1) (Mishra et al., 2000; Shindy, 2012; Shindy, 2017). The heterocyclic structure can be pyrrole, imidazole, thiazole, benzothiazole, pyridine, or quinoline (Shindy, 2017; Gopika et al., 2021). Variations in the polymethine chain and nitrogen substituents on the heterocycles cause structural diversity in cyanine derivatives. The bridging polymethine chain is a conjugated chain formed by sp^2^ hybrid carbon atoms ending with a positive charge on the nitrogen through the displacement of π-electrons (Gopika et al., 2021). The cyanine family is very large; thus, subclassification is needed for its conjugates. Based on the terminal groups in the structure, they can be classified as closed chain cyanines, streptocyanines, merocyanines, squarylium cyanines, and hemicyanines (Figure 1) (Shindy, 2017; Gopika et al., 2021). Cyanine dyes can also be subcategorized based on the number of bridging methine chain as apocyanines (directly linked without any methine), monomethine cyanine, trimethine cyanine, pentamethine cyanine (dicarboxycyanine), and heptamethine cyanine (tricarbocyanine) (Shindy, 2012). Streptocyanines contain a non-heterocyclic aromatic moiety at both ends, while merocyanine dyes have an amino group in one end and a carbonyl group in other end, which are connected *via* polymethine chain (Kulinich and Ishchenko, 2009; León et al., 2016). Squarylium cyanines have a squaraine moiety in the center of the π-conjugation (Ferreira et al., 2013). Hemicyanine scaffolds feature a donor-π−acceptor (D-π−Α) system as their structures have a terminal hydroxyl, alkoxy, or amino group as the electron donor and a nitrogen heterocyclic moiety with a positive charge as the electron acceptor group that are connected *via* a conjugated chain (Jędrzejewska et al., 2003). Cyanines usually exist in neutral and zwitterionic resonance forms with cationic and anionic moieties coexisting in the molecule. The absorption and emission maxima of cyanine dyes are affected by the polymethine bridge length as conjugated carbon chain with hetereocyclic units at the terminal position forces the formation of a linear shape (Shindy, 2017).

Cyanine core is among the most investigated fluorophores due to its favorable optical properties such as high molar absorptivity, narrow absorption/emission bands, reasonable fluorescence quantum yield, and most importantly, tunable fluorescence profile from the UV–vis to NIR range (Delaey et al., 2000; Mishra et al., 2000; Kulbacka et al., 2011; Bhattarai and Dai, 2017; Li Y. et al., 2020; Li et al., 2021). Additionally, the excellent biocompatibility and low toxicity make them a great candidate for biological studies spanning from imaging to therapy. Absorption maxima of cyanine derivatives can be tuned to the NIR region (650–900 nm) through easy structural modifications thanks to well-established cyanine chemistry. This is critical since NIR light is most suitable for biological applications as it provides deeper tissue penetration and light safety (Mallidi et al., 2016).

**FIGURE 2:**
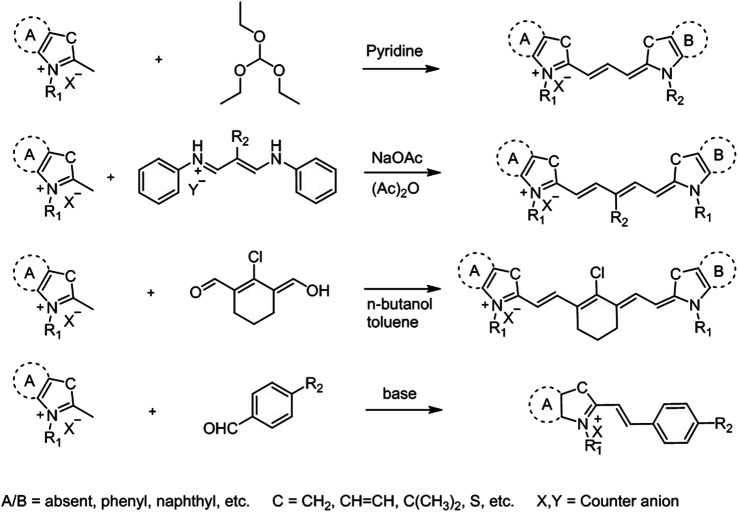
General synthetic approaches for cyanine derivatives (Li et al., 1997).

To that end, significant efforts have been put to develop effective methods for the synthesis of cyanine dyes and to improve their photophysical properties for biological applications (Figure 2) (Narayanan and Patonay, 1995; Li et al., 1997; Jędrzejewska et al., 2003; Kulinich and Ishchenko, 2009). The most popular method for the synthesis of cyanine dyes is the condensation of aromatic quaternary ammonium salts with different reagents in the presence of a base. For example, trimethine cyanine can be obtained through condensation of quaternary ammonium salts with trimethyl orthoformate resulting in different trimethine cyanine fluorophores depending on the -R groups in the heterocycle. General strategy for pentamethine cyanine synthesis is the condensation of quaternary ammonium salts with bis-aniline–conjugated polymethine chain bearing different *meso*-substituents. Heptamethine–cyanine dyes with *meso*-chlorine substitution and related heptamethine–cyanine dyes without substitution on the vinyl chain can be synthesized using condensation of quaternary indolium salts as shown in Figure 2 (Li et al., 1997). Additionally, hemicyanine-like structures can be obtained by the condensation of heterocyclic bases (Fischer’s base) containing activated methyl groups with unsaturated benzaldehydes (Jędrzejewska et al., 2003).

Cyanine fluorophores, especially those excited with a light in the NIR, are studied for both fluorescence imaging and phototherapeutic effect, which renders them as phototheranostic agents (Jung et al., 2018; Chen et al., 2019; Li et al., 2021). However, the phototherapeutic efficacy of conventional cyanine dyes is not adequate due to low photoconversion efficiency, poor photostability, and undesired aggregation in aqueous solution. Therefore, a number of studies have been carried to improve these properties, including conjugation with other therapeutic agents to enhance its therapeutic efficacy and broaden its therapeutic modality (Zhao et al., 2019; Ebaston et al., 2021; Li et al., 2021). For the phototherapeutic applications, especially PDT, improvement of ^1^O_2_ generation efficiency is usually achieved by introduction of heavy atoms (iodine, bromine, selenium, etc.) to favor ISC as a result of enhanced spin-orbit coupling (Santos et al., 2005; Atchison et al., 2017; Cao et al., 2019; Ebaston et al., 2021). For example, iodination on the heterocyclic core in heptamethine cyanine increases the ISC efficiency through heavy-atom effect with resulting cultivated therapeutic efficacy (Shi et al., 2016; Huang and Luo, 2020). On the other hand, it is also possible to achieve high singlet oxygen yield with heavy-atom–free cyanine conjugates by incorporating them with an ISC-promoting moiety such as tetramethylpiperidinyloxy (TEMPO) (Yang et al., 2020).

Another issue with the cyanine core is its chemical instability under light irradiation. So far, although photoconversion of cyanine fluorophores has been known, the underlying mechanism and the species involved in these photoconversion reactions are still unclear (Shindy, 2017). A recent study by Schnermann’s group showed that heptamethine cyanines are converted into pentamethine cyanines, which, in turn, are converted to trimethine cyanines through light irradiation (Matikonda et al., 2021).

Some other common limiting factors such as low water solubility and ease of self-aggregation have been tried to be solved by introducing hydrophilic units (e.g., sulfonate groups) to the nitrogen-containing heterocycles. In this direction, a number of phototheranostic agents based on cyanine core with improved water solubility and therapeutic efficiency have been developed successfully so far (Luo et al., 2016; Li S. et al., 2017; Huang and Luo, 2020; Gunduz et al., 2021).

### Cyanine-Based Photosensitizers in Phototherapies

**FIGURE 3:**
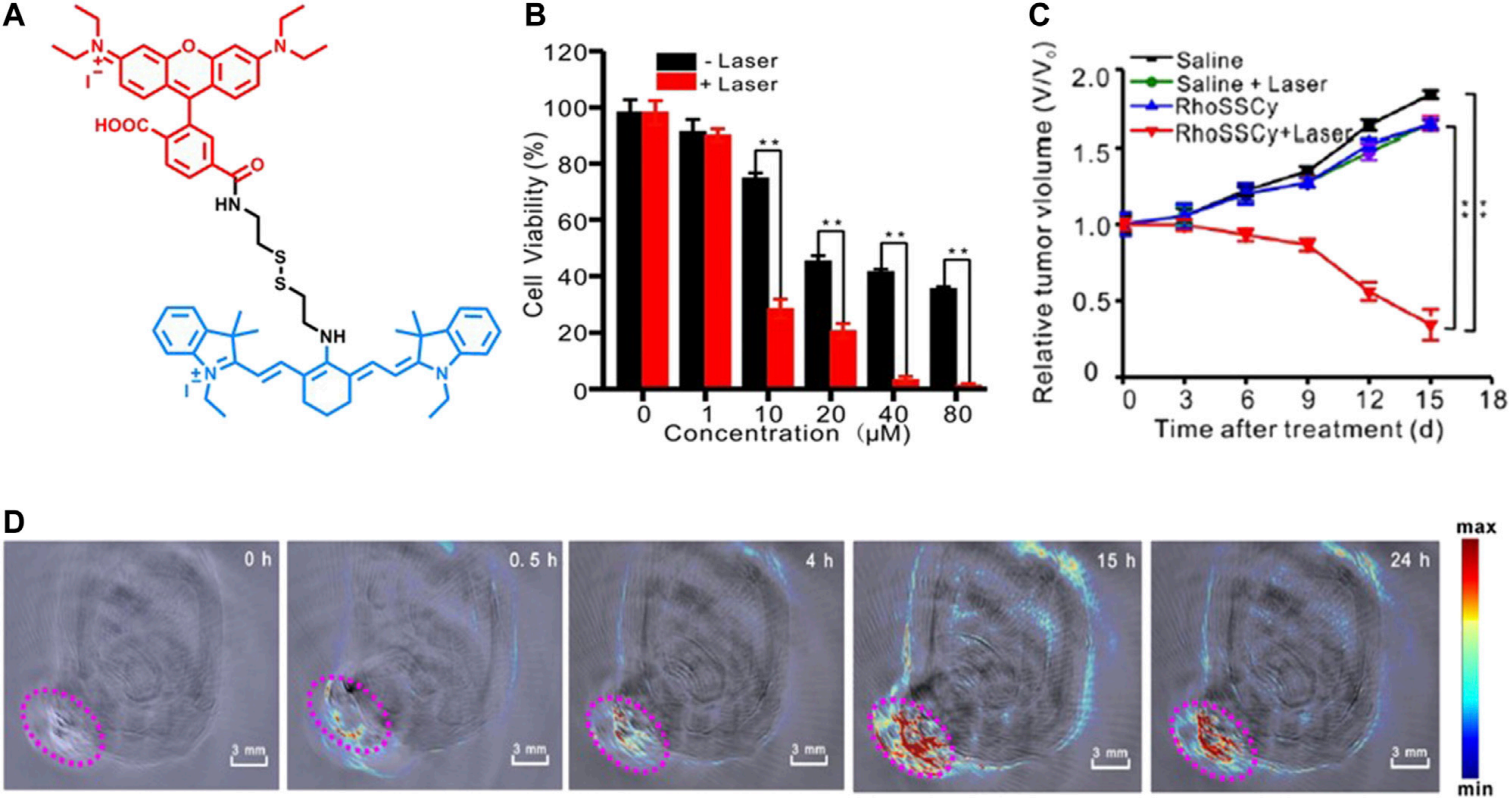
**(A)** Representative structure of RhoSSCy, **(B)** the effect of RhoSSCy-induced PDT on MCF7 cell viability with different PS concentrations, **(C)** the tumor growth of MCF7 xenograft tumors after different treatments within 15 days, and **(D)** PA imaging of a tumor-bearing nude mouse at different hours. (Reproduced from ref. Meng et al. (2017b) with permission from Ivyspring, copyright 2017.)

Meng et al. developed a small molecule probe RhoSSCy (Figure 3A) by conjugating 5ʹcarboxyrhodamines (Rho) and IR765, which offers thiol/pH dual-sensing, tumor-targeting, NIRF/photoacoustic (PA) dual-modal imaging, and PDT potential. The fluorescence response of RhoSSCy was evaluated on MCF7 cells, and a strong fluorescence was observed in the presence of GSH at low pH (pH = 6). PA imaging revealed that RhoSSCy accumulated in tumor tissue in 4 h and reached its maximum in 15 h in MCF7 cells (Figure 3D). The irradiation of RhoSSCy at 660-nm laser wavelength (30 mW/cm^2^) for 5 min caused significant cell death at 10 µM and almost complete cell death at 40 µM (Figure 3B). A significant tumor suppression was observed after two weeks of irradiation at 100 mW/cm^2^ (Figure 3C) (Meng et al., 2017b).

**FIGURE 4:**
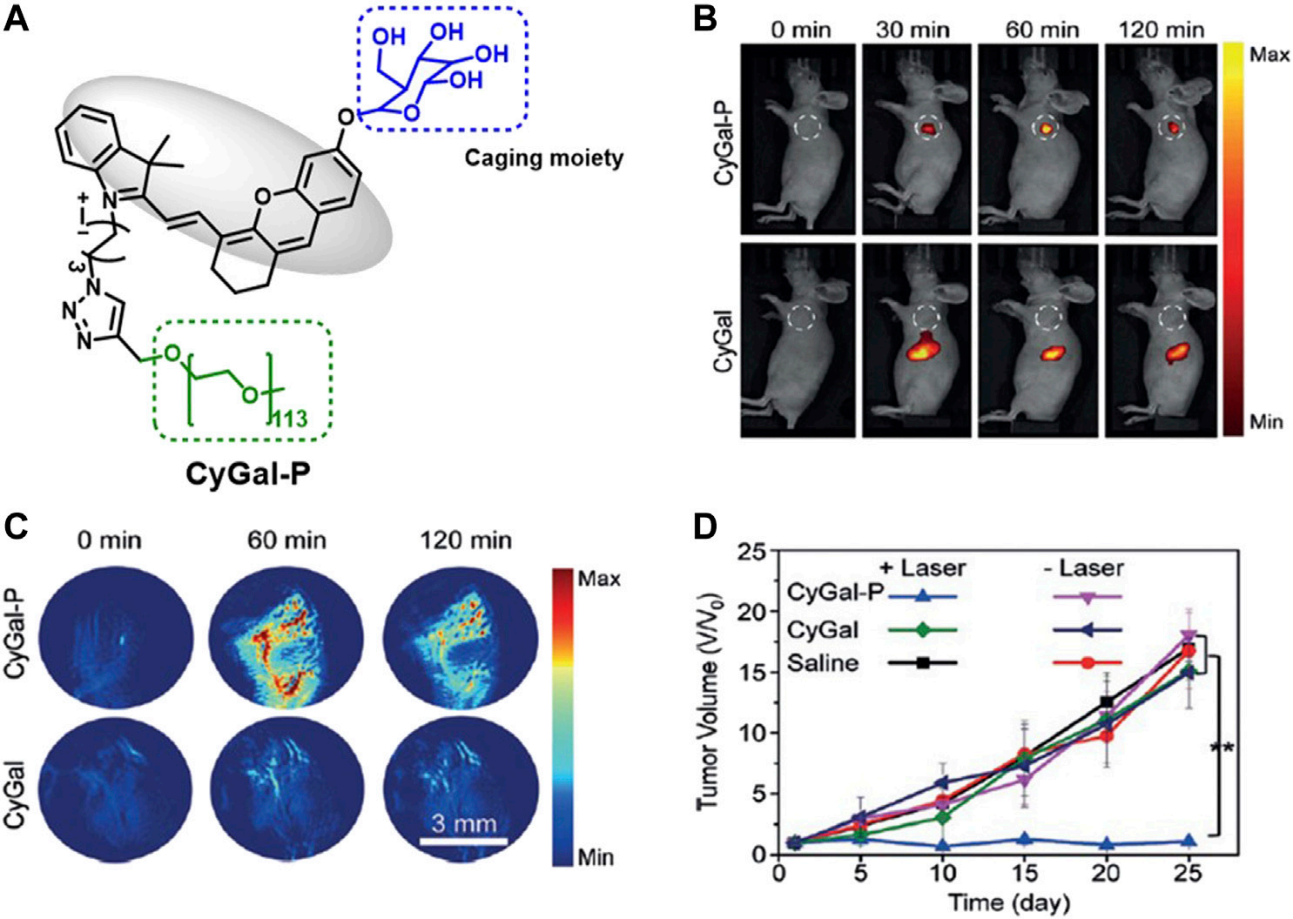
**(A)** Representative structure of CyGal-P, **(B)** fluorescence, and **(C)** PA images of SKOV3 tumor–bearing living mice after intravenous injection of CyGal and CyGal-P at different times at 300 µM, and **(D)** tumor growth in SKOV3 tumor–bearing mice after laser irradiation of CyGal and CyGal-P. (Reproduced from ref. Zhen et al. (2018) with permission from Wiley, copyright 2018.)

Zhen et al. developed a galactose-caged NIR hemicyanine dye (CyGal-P), which is linked with a PEG chain, for image-guided cancer therapy and compared its potential with parent CyGal core (without PEG) on ovarian cancer, where βGal is known to be overexpressed Figure 4. The irradiation of CyGal-P with 680-nm laser wavelength (0.6 W/cm^2^) for 5 min caused almost complete cell death on SKOV3 cell line as a result of PTT action, while there was no cell death after irradiation of CyGal. The *in vivo* NIRF and photoacoustic images showed that CyGal-P accumulated in the tumor in 60 min; however, the accumulation of CyGal was very low in the tumor region. In *in vivo* animal models, complete tumor elimination was achieved without any abnormalities (Zhen et al., 2018).

The PDT efficacy of mitochondria-targeted NIR CYBF_2_, a boron difluoride complex–modified cyanine, was evaluated on MCF7 cells based on various concentrations and light doses. CYBF_2_-treated MCF7 cells showed strong fluorescence after 10 min, suggesting quick internalization of CYBF_2_ by cancer cells. The IC_50_ value was found to be 2 µM under 660-nm laser irradiation (90 J/cm^2^ light dose), and enhanced toxicity was observed with higher light doses and agent concentrations. The irradiation of CYBF_2_ provided cell death by causing severe mitochondrial depolarization (Zhao et al., 2019).

**FIGURE 5:**
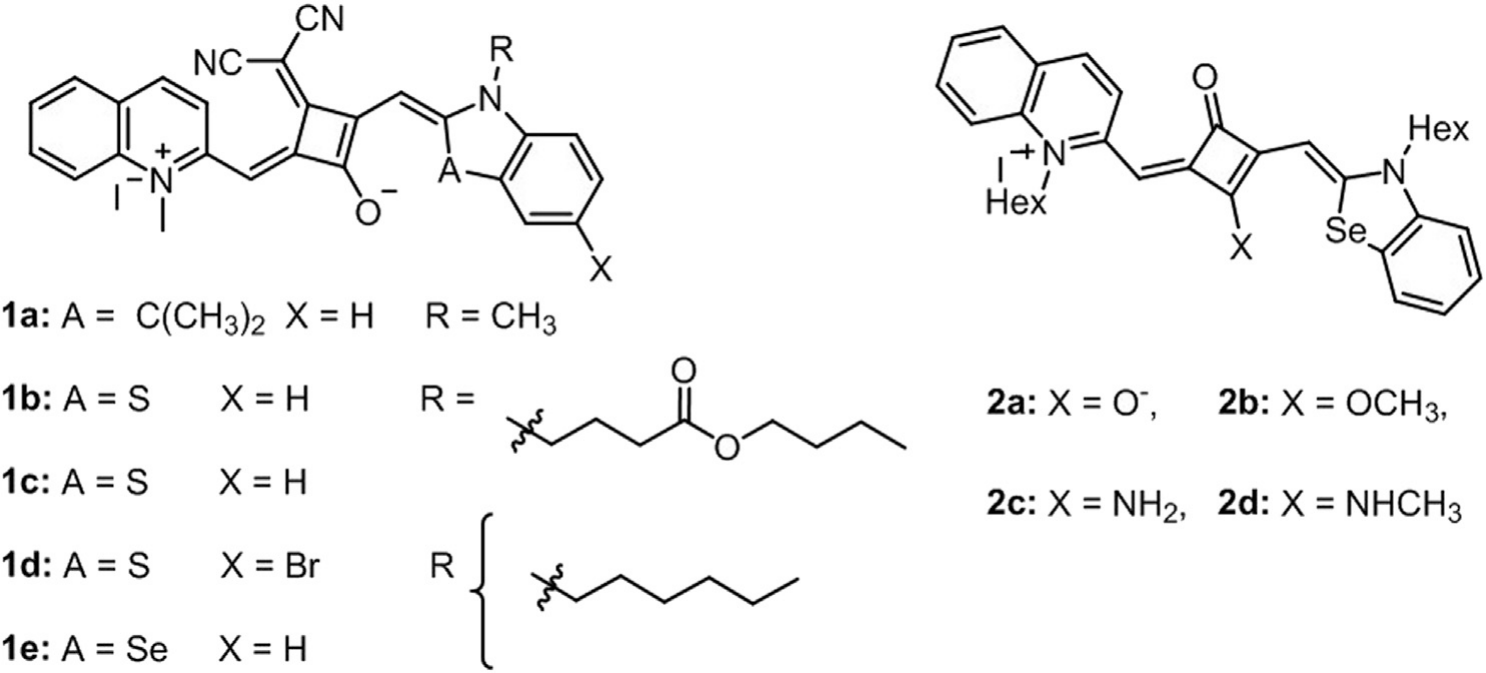
Representative structures of dyes in **(A)** D Martins et al. (2020) and **(B)** Lima et al. (2020).

Reis et al. introduced two articles on photophysical and photochemical properties and the photobiological activity of squaraine cyanine dyes (1a, b, c, d, and e) (Figure 5A). These dyes exhibited sharp absorption in the NIR region in several organic solvents and broad absorption between 600–800 nm in aqueous buffered solutions (PBS and DMEM). Singlet oxygen quantum yield of dyes were reported within the range of 0.01–0.08 in chloroform with moderate light stability. Furthermore, a cell viability assay was performed on human colorectal adenocarcinoma (Caco-2) and human hepatocellular carcinoma (HepG2) to show *in vitro* photocytotoxicity activity. It was reported that 1a, 1c, and 1e displayed comparably higher cytotoxicity with IC_50_ value below 0.5 μM in both cell lines (D Martins et al., 2020). In a follow-up study, Reis’s group developed a series of quinoline- and benzoselenazole-derived unsymmetrical squaraine cyanine dyes (2a, b, c, and d) (Figure 5B). Similarly, these dyes have sharp absorption band between 636 and 733 nm in organic solvents, and broader signals in aqueous solutions associated with the formation of aggregates and low singlet oxygen yield. *In vitro* phototoxicity of dyes was proven in breast cancer cell lines BT-474 (human ductal breast carcinoma) and MCF7 (human breast adenocarcinoma derived from pleural effusion) with reasonable IC_50_ values (up to 0.6 μM). As an improvement, these dyes were found to be photostable, highlighting the stability of the zwitterionic dyes and improved the singlet oxygen quantum yields (Lima et al., 2020).

**FIGURE 6:**
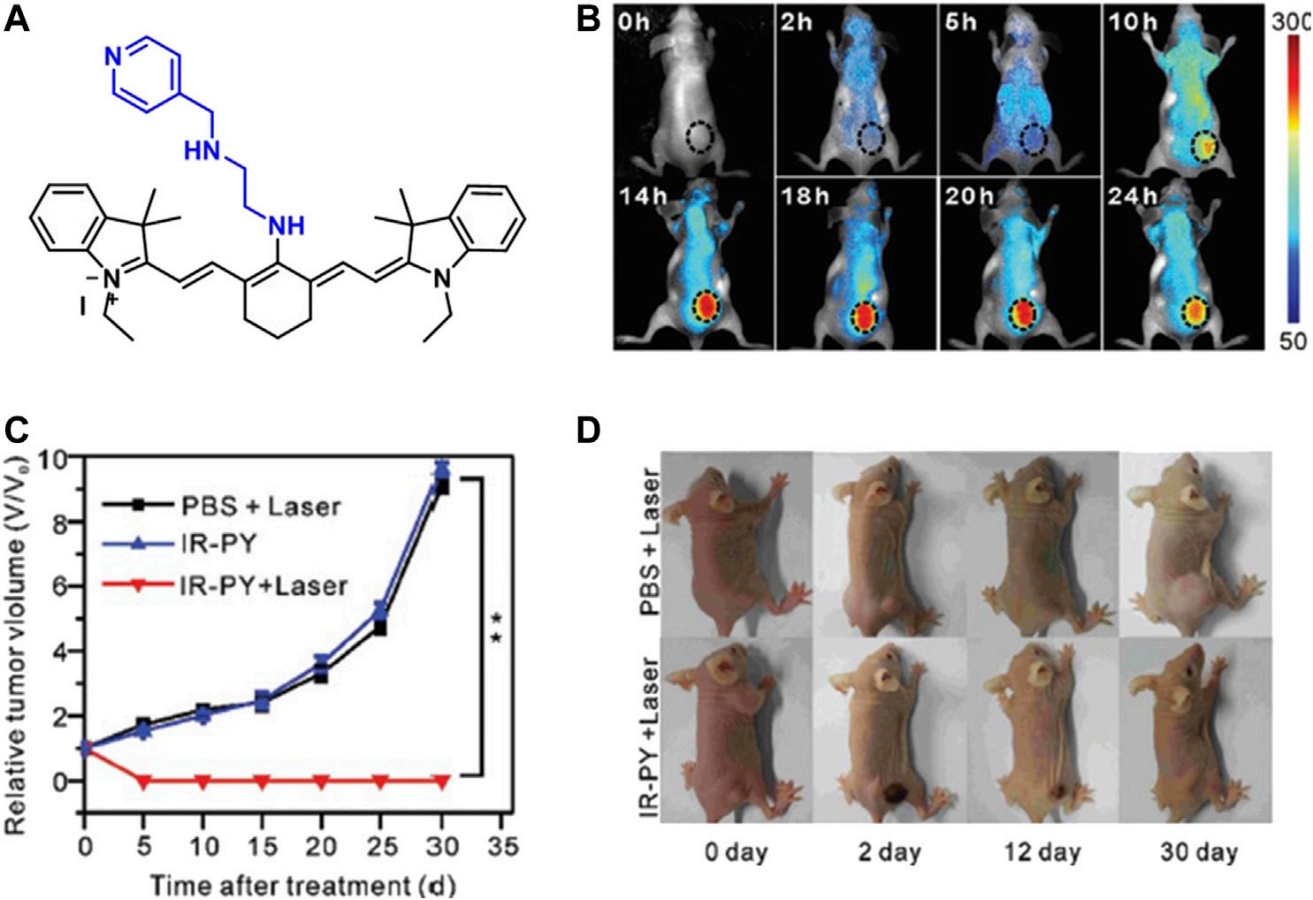
**(A)** Structure of IR-PY, **(B)** NIRF images of MCF7 tumor–bearing living nude mice after injection of IR-PY at different times, **(C)** the tumor growth, and **(D)** representative images of mice bearing after different treatments. (Reproduced from ref. Meng et al. (2017a) with permission from RSC, copyright 2017.)

An NIR fluorescent heptamethine cyanine dye, IR-822, with a high extinction coefficient was conjugated with a pH-sensitive receptor N^1^-(pyridin-4-ylmethyl)ethane-1,2-diamine (PY) to increase its tumor accumulation and imaging capability. This molecular probe (Figure 6A) was designed to work as a switch-on in the acidic tumor microenvironment which allowed the enhancement of the NIR imaging *in vivo*. The conjugate had a strong absorption in the NIR, and a peak shifted from 637 to 792 nm with increasing pH value, while showing a good photothermal effect. The probe was tested on MCF7 cells and after incubation for 24 h, no significant cytotoxicity was observed up to 100 μg/ml concentration. The flow cytometry revealed that the fluorescence signal from the IR-PY reached its maximum value just after 4 min. The targeting effect was investigated with both healthy and cancerous liver (Lo2 and HepG2), lung (MRC-5 and A549), kidney (293 and A498), and breast (MCF-10A and MCF7) cell lines, and the data indicated that the cellular uptake was significantly higher in the tumor cell lines. Finally, they examined the *in vivo* tumor targeting and NIR imaging with tumor-bearing nude mice upon addition of 40 μg/ml IR-PY. After 10 h of injection, the mice showed strong NIR fluorescence signal on the tumor tissue and after 14 h, the fluorescence signal reached a plateau (Figure 6B). At 14-h time point, the mice were further treated with a low-powered NIR laser (808 nm, 0.4 W/cm^2^) for 3 min, and it was shown that tumors in mice treated with IR-PY plus laser exhibited stronger hemorrhagic injury and severe necrotic tissue than the control group with no tumor recurrence for 30 days after treatment (Figures 6C,D) (Meng et al., 2017a).

**FIGURE 7:**
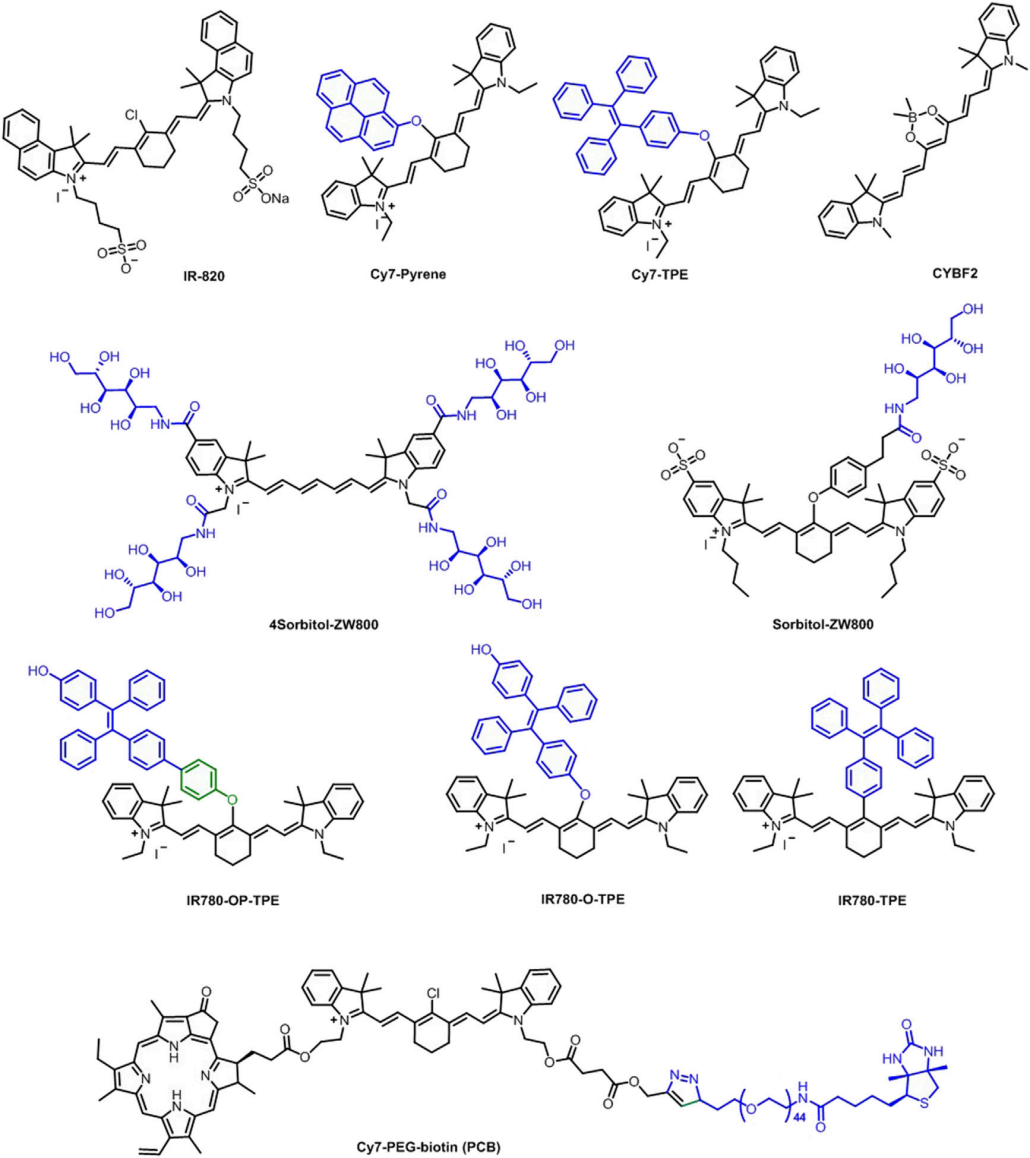
Heavy atom–free cyanine-based phototherapy agents.

Benefiting from the strong absorption and broad NIR emission (700–1,300 nm) of IR-820, Feng et al. developed an excretable NIR-II imaging and NIR-I PTT probe using IR-820 for cardiovascular imaging and therapy (Figure 7) **(3)**. The probe was utilized without any surface modification *in vivo* by intravenous injection of 0.5 mg/ml dose. The data showed good biocompatibility by clearing out from the liver in 5 weeks and NIR-II fluorescence imaging–guided photothermal therapy on the subcutaneous tumor of mice. Additionally, *in vivo* cerebrovascular imaging of mice was conducted, achieving high spatial resolution (6.061 μm) even at the depth of ∼800 μm (Feng et al., 2019).

Using high targeting ability of the sorbitol moiety, a bifunctional heptamethine cyanine fluorophore, sorbitol–ZW800 conjugate, was developed as a PTT agent for *in vivo* cancer imaging and therapy (Figure 7). A temperature increase of 58.7°C was observed after the NIR laser irradiation (1.1 W/cm^2^) for 5 min. The photothermal conversion efficiency was calculated as 32.6% which was deemed to be relatively higher than many inorganic phototherapy agents. *In vivo* studies were conducted with HT-29 tumor–bearing mice using a single dose (10 nmol, 0.4 mg/kg) of injection. Fluorescence signal on the tumor tissue reached its maximum value in 2 h and gradually decreased, showing good imaging potential. Additionally, *in vivo* PTT effect was assessed by treating the tumor-bearing mice with 10 nmol 0.4 mg/kg sorbitol–ZW800 conjugate 2 h prior to laser irradiation at 808-nm laser (1.1 W/cm^2^) exposure for 5 min. A 58.7°C temperature increase was observed at the tumor site 5 min after laser irradiation, proving a strong PTT potential (Lee et al., 2019).

Another sorbitol-conjugated NIR fluorophore (4Sorbitol-800) was designed by Yoo et al. to demonstrate tumor-targeting ability of sorbitol moieties and high PTT properties of heptamethine cyanine core (Figure 7). The temperature was elevated to 81.0°C in 4Sorbitol-800 solution (100 μM) when irradiated with an 808 nm NIR laser (1.1 W/cm^2^) for 1 min. The photothermal conversion efficiency was calculated as 30.5%. Additionally, *in vitro* PTT studies showed that it can produce efficient hyperthermia with NIR laser irradiation. Finally, HT-29 tumor–bearing mice were treated intravenously with the conjugate and the fluorescence signals were tracked for 24 h after injection. The signal peaked at 2 h and gradually decreased within the next 4 h. The biodistribution was confirmed from renal and hepatic clearance at 4 and 24 h postinjection without significant uptake in other tissues. The *in vivo* PTT efficacy was evaluated by injecting 4Sorbitol-800 (10 nmol, 0.48 mg/kg) into mice 2 h before laser treatment (808 nm laser at 1.1 W/cm^2^ for 5 min) and a 57.4°C temperature increase was observed. Treated mice showed no tumor recurrence for 7 days (Yoo et al., 2020).

The photostability problem and self-quenching property of cyanine-based photosensitizers can be minimized by incorporating an aggregation-induced emission (AIE) character to the PS. In this direction, Zhao et al. attached an AIE unit, namely, tetraphenylethene (TPE) into the structure of heptamethine cyanine IR780, which normally suffers from aggregation-caused quenching effect in high concentrations. Three different TPE-IR780 derivatives (IR780-OP-TPE, IR780-O-TPE, and IR780-TPE) (Figure 7) *via* different linkage groups were synthesized and their properties were investigated. Because of its poor dispersibility in culture medium, IR780-TPE was not investigated in the cell studies. HeLa cells were incubated with both probes, and it was observed that the cells were “light-up” in 5 min of incubation, showing the ability for fluorescent imaging. The mitochondrial targeting ability of the conjugates were assessed using a mitochondrial-tracker green (MTG) dye, and results showed that the NIR signal from the probes overlapped with the green signal of the tracker. Furthermore, IR780-O-TPE was found to have a better photostability due to its enhanced aggregation in mitochondria. *In vitro* studies showed that the two variations of IR780 were more toxic to the cancer cells than normal cells, proving a potential to be used as selective therapeutic agents toward cancer cells. The PTT effect was then investigated under irradiation by an 808-nm laser at 0.5 W/cm^2^ for 10 min and 10 and 15°C temperature increase were recorded for IR780 and the three IR780 conjugates, respectively, at 50 μM concentration. Late apoptotic cell death increased when the cells were treated with the probes (0.6 μM, 24 h) upon 5-min irradiation. Furthermore, in 4T1 tumor-bearing mice, IR780-O-TPE was proved to have a more effective PTT response than IR780 itself, proposing an optimal photosensitizer (Zhao et al., 2020).

Wu et al*.* synthesized two agents, namely, Cy7−pyrene and Cy7−TPE (Figure 7), by conjugating pyrene and tetraphenylethylene (TPE) moieties on Cy backbones to form well-organized H-aggregates in aqueous solutions toward achieving high PTT efficiency. H-aggregates of the fluorophores quench the fluorescence signal as it favors nonradiative transitions, which may result in PTT. This phenomenon is well studied in this work by introducing pyrene that is known to form strong π−π stacking, resulting in H-aggregation. However, the introduction of the TPE group, which was expected to inhibit π−π stacking and H-aggregation, was also shown to induce H-aggregation without π−π stacking and resulted in PTT effect. The photothermal conversion efficiency was calculated as 9.5 and 22.3% for Cy7-TPE and Cy7-pyrene, respectively. *In vitro* cytotoxicity was evaluated using HeLa cells and up to 100 μM concentration was determined as nontoxic and biocompatible. With an 808-nm laser irradiation for 15 min, almost all cells were dead. NIR fluorescence imaging–guided therapy was investigated using 4T1 tumor–bearing mice. Based on the data for the fluorescence signal from the tumors, 8–24 h after the injection was determined as the most suitable time range for the PTT. Furthermore, the mice were treated with an 808-nm laser for 15 min 8–24 h after the injection, and the real-time surface temperature of the tumors was monitored using IR thermal camera. Both the Cy7-pyrene and Cy7-TPE heated up to 60°C in less than 10 min of irradiation and in both groups, tumors were completely ablated (Wu et al., 2020).

In another study, hydrophobic pyropheophorbide A (Ppa) was covalently conjugated to Cy7-PEG-biotin (PCB) for image-guided PTT and PDT (Figure 7). The release of Ppa from PCB was studied in PBS and the total release of Ppa in PBS was less than 8% after 2 days at pH = 7.4. However, the irradiation of PCB at 808 nm provided 80% release in the first 10 h. On the other hand, there was a little increase in release after 670 nm irradiation. Single 808 nm (0.5 W/cm^2^, 6 min) and 670 nm (20 mW/cm^2^, 6 min) irradiations caused limited cell death on HepG2 cells, while combined PTT + PDT and PDT + PTT provided a significant cell death under same conditions. In addition, a combination of PTT + PDT provided more cell death than PDT + PTT due to high release of Ppa. The combination of PTT + PDT ablated tumor effectively compared to PDT + PTT (Xue et al., 2019).

Although cyanine derivatives have been utilized in PDT and synergistic PDT + PTT applications, they have very low singlet oxygen quantum yields, which causes inadequate efficacy in PDT and PDT + PTT actions. Therefore, phototherapies with cyanine dyes require high drug doses or high laser irradiation or long irradiation time for efficient results. Decorating cyanine cores with heavy atoms appeared as a promising solution to increase singlet oxygen production, improving spin–orbit coupling–mediated intersystem crossing (ISC).

### Heavy Atom–Modified Cyanine Dyes in Phototherapies

**FIGURE 8:**
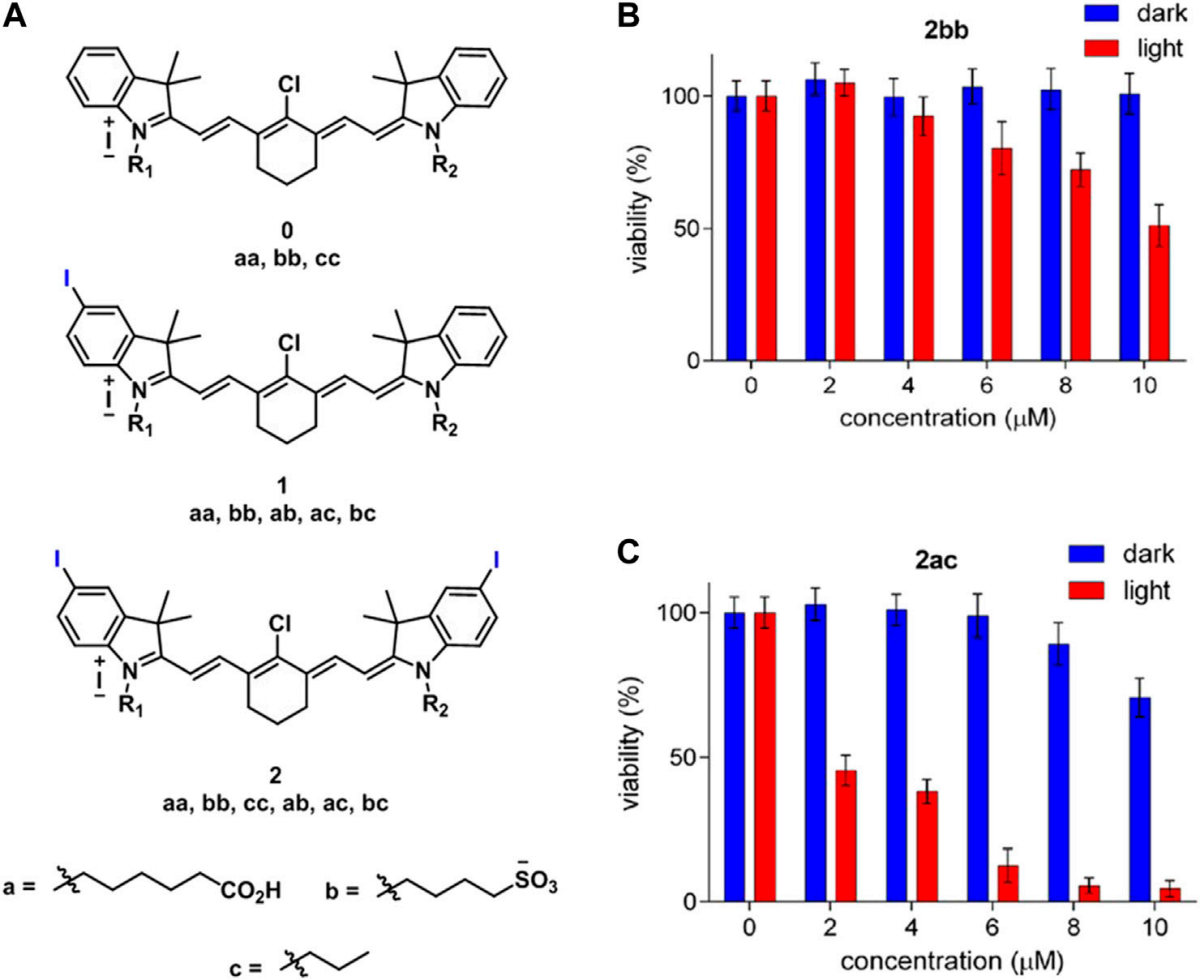
**(A)** Structure of the cyanines derivatives (0aa, bb, cc, 1aa, bb, ab, ac, bc, 2aa, bb, cc, ac, and bc), toxicity of **(B)** 2bb and **(C)** 2ac on HepG2 cells with and without laser irradiation. (Reproduced from ref. Usama et al. (2018) with permission from ACS, copyright 2018.)

Usama et al. developed 10 different near-IR fluorescent dyes, which contain zero, one, or two iodine atoms for photodynamic therapy under 780 nm irradiation and compared their potential with 2bb (literature control) (Figure 8A). The prepared compounds 2aa, 2ab, 2ac, and 2bc (Figure 8A) showed improved toxicity compared to 2bb after 10 min irradiation at 3.8 mW/cm^2^ on HepG2 cells. More specifically, 2ac provided almost complete cell death at 10 µM, while 2bb caused less than 50% cell death (Figures 8B,C). The enhanced cytotoxicity occurred as a result of accumulation of these novel sensitizers in mitochondria. On the other side, 2bb was known to be accumulated in lysosome (Usama et al., 2018).

**FIGURE 9:**
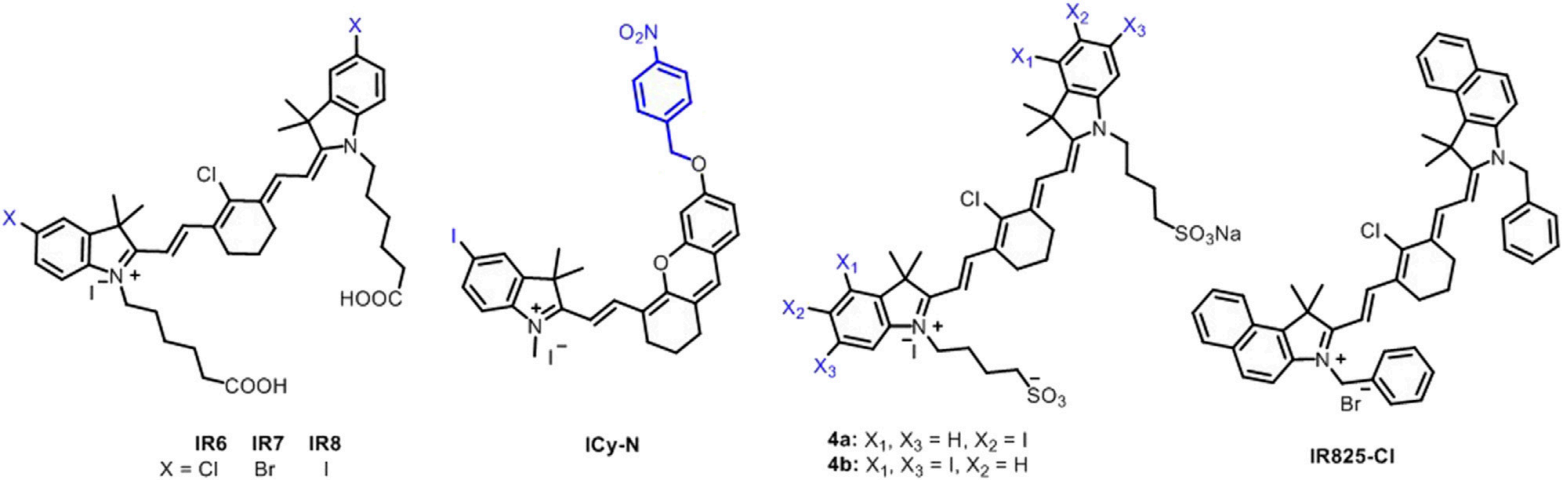
Heavy atom–modified cyanine-based phototherapy agents.

The effect of halogens (chlorine, bromine, and iodine) on phototherapy potential of heptamethine dye was evaluated on HeLa cells. All three halogenated dyes IR6 (chlorine), IR7 (bromine), and IR8 (iodine) showed high singlet oxygen capacity and photothermal conversion efficiency (Figure 9). The photothermal conversion efficiency was 42.3, 43.4,, and 46.6% for IR6, IR7, and IR8, respectively. IR8 showed enhanced photostability compared to IR6 and IR7 after multiple laser irradiations; however, there was still 8.7°C temperature loss between the first and last cycles. In addition, IR8 provided more cell death than IR6 and IR7 after 5-min laser irradiation of 808 nm at 1 W/cm^2^ having IC_50_ value at 16.2 μg/ml (Liu et al., 2021). NIR hemicyanine dye ICy-N was developed by modifying Cy-OH with 4-nitrobenzyl bromide and iodine for hypoxia imaging and PDT (Figure 9). ICy-N has no fluorescence and low singlet oxygen quantum yield; however, the reduced product, ICy-OH, showed high singlet oxygen quantum yield and fluorescence potential, which occurred as a result of reduction of ICy-N by nitroreductase (NTR). 2.5 µM ICy-N–treated 4T1 cells exhibited no fluorescence under normoxic conditions, while it induced strong fluorescence under hypoxia (10%, 2%, and <1% O_2_) due to enhanced overexpression of NTR under hypoxic conditions. Irradiation of ICy-N (1.5 mM) with a 660-nm laser irradiation at 12 mW/cm^2^ for 5 min caused 20 and 80% cell death under 21 and 10% O_2_ conditions, respectively. *In vivo*, there was no growth in tumor volume after the irradiation of ICy-N at 100 mW/cm^2^ for 20 min, while a 5-fold increase was observed with only laser or ICy-N treatment (Xu et al., 2019).

**FIGURE 10:**
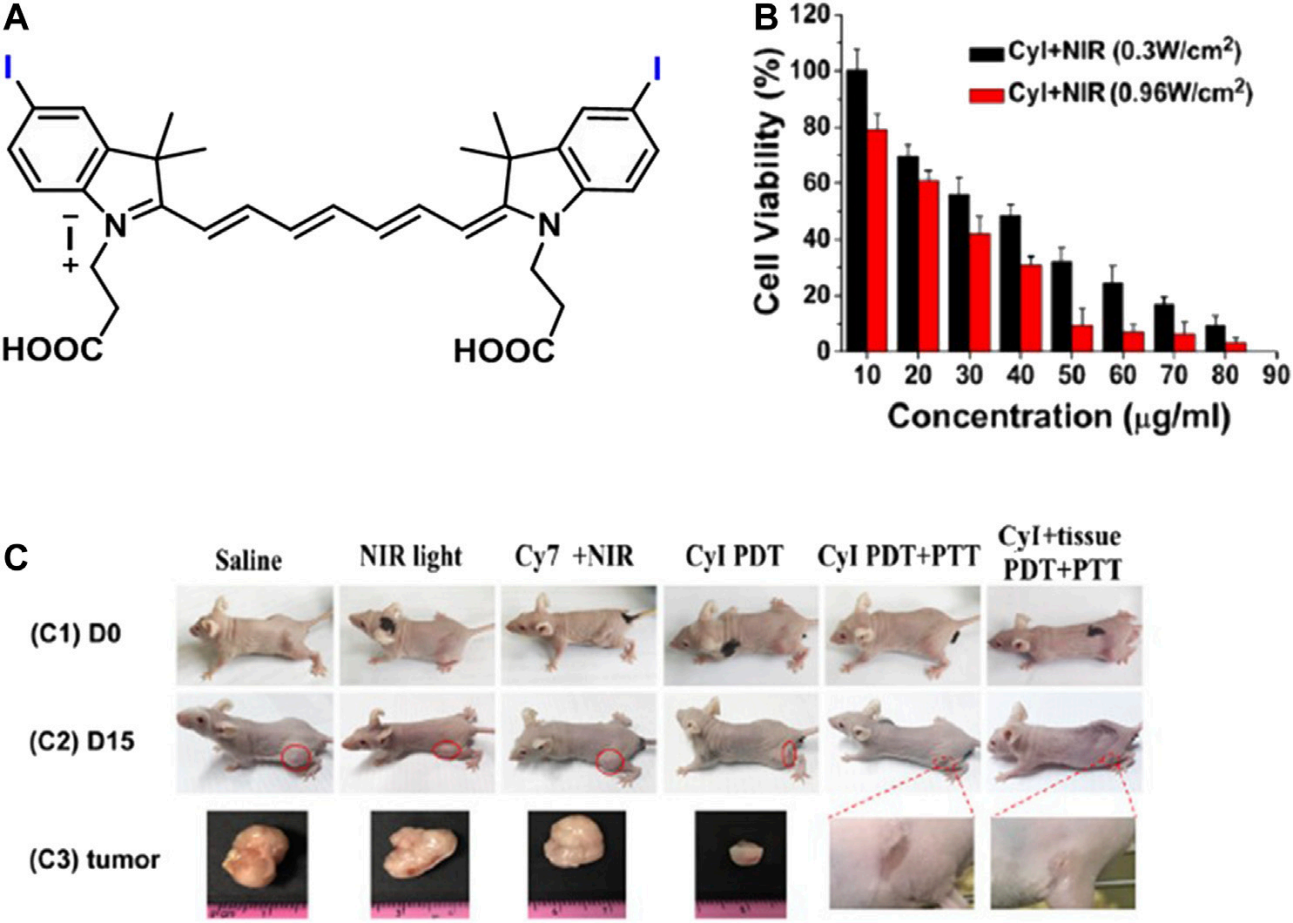
**(A)** Representative structure of CyI, **(B)** the effect of PDT (0.3 W/cm^2^) and PTT/PDT (0.96 W/cm^2^) on CyI-treated HepG2 cells, and **(C)** images of mice before treatment (C1) and after different treatments (C2) and tumors after treatments (C3) in 15 days. (Reproduced from ref. Cao et al. (2019) with permission from ACS, copyright 2019.)

Atchison et al. evaluated PDT efficiency of two iodinated derivatives of IR-783, di-iodinated (4a) or tetraiodinated (4b) in BxPC-3 and MIA-PaCa-2 pancreatic cells, and Ectopic BxPC-3 Luc pancreatic tumor after 1-min irradiation of 780-nm laser wavelength at 100 mW (Figure 9). There was no difference between 6a and 6b in BxPC-3; however, 6a provided more cell deaths in MIA-PaCa-2. 6a induced more than 90 and 60% cell death in MIA-PaCa-2 and BxPC-3, respectively, at 50 µM. The irradiation of 6a for 9 min caused almost total tumor elimination *in vivo*. However, a 39% increase was observed in the tumor at day 11 (Atchison et al., 2017). A mitochondrial cyanine dye IR825-Cl was developed for red fluorescence imaging and PTT with 17.4% photothermal conversion efficiency (Figure 9). It induced 70% and more than 95% cell mortality at 5 and 8–20 μg/ml under an 808-nm laser irradiation at 1 W/cm^2^ for 5 min on HeLa cells mostly through late apoptosis/necrosis (Pan et al., 2017). Synergistic PTT/PDT with iodine-modified NIR dye CyI (Figure 10A) resulted in 95.42 ± 8.1% cell death in HepG2 cells after 1-min irradiation of 808 nm at 0.96 W/cm^2^, while PDT alone induced only 64.4 ± 5.9% at 0.3 W/cm^2^ (Figure 10B). *In vivo*, the total inhibition rate was 100% and 39.8% after synergistic PTT/PDT and PDT alone, respectively. The total inhibition rate was still 100% after synergistic PTT/PDT when the tumor was covered with 1-cm pork tissues (Figure 10C) (Cao et al., 2019).

**FIGURE 11:**
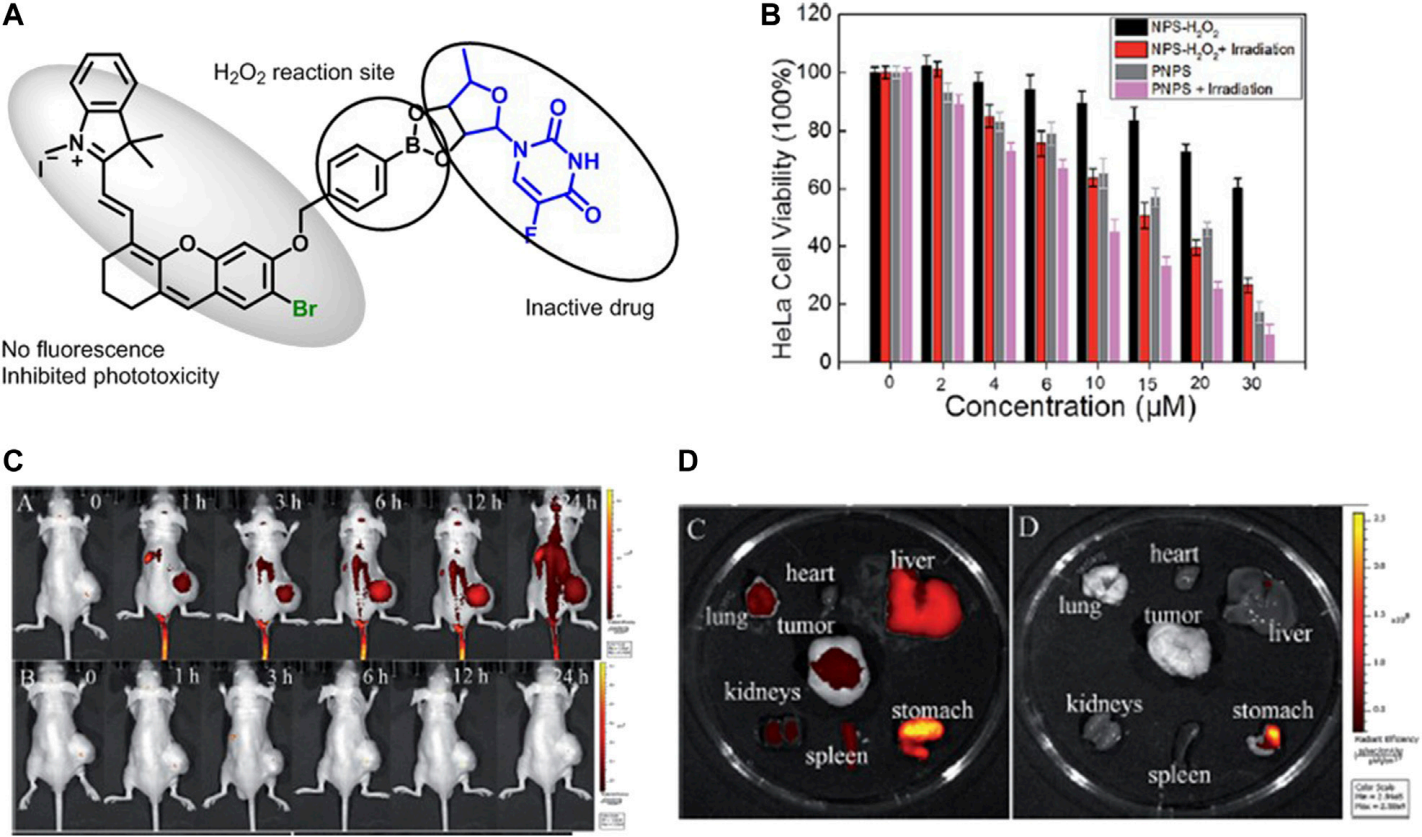
**(A)** Representative design of PNPS, **(B)** the viability of HeLa cells after PNPS and PNPS + laser treatment, **(C)** *in vivo* imaging of HCT116 tumor–bearing mice after injection of saline and PNPS at different times, and **(D)** fluorescence images of different organs after injection of PNPS and saline at 25 h. (Reproduced from ref. Liu et al. (2017) with permission from RSC, copyright 2017.)

Liu et al*.* developed a mitochondria-targeted prodrug (PNPS) (Figure 11A) by conjugating 5ʹ-deoxy-5-fluorouridine (5ʹ-DFUR) and brominated hemicyanine for NIR imaging–guided and synergetic photodynamic-chemotherapy. A prodrug PNPS showed improved toxicity compared to commercial 5ʹ-DFUR on HeLa and HepG2 cells. In addition, the irradiation of PNPS under white light (50 W) reduced IC_50_ value from 16.6 and 14.8 µM to 9.32 and 8.15 µM for HeLa (Figure 11B) and HepG2 cells, respectively. PNPS-treated HCT116 tumor–bearing mice showed NIR fluorescence in the tumor after 1 h injection, indicating the release of activated drug (Liu et al., 2017) (Figures 9D, 11C).

**FIGURE 12:**
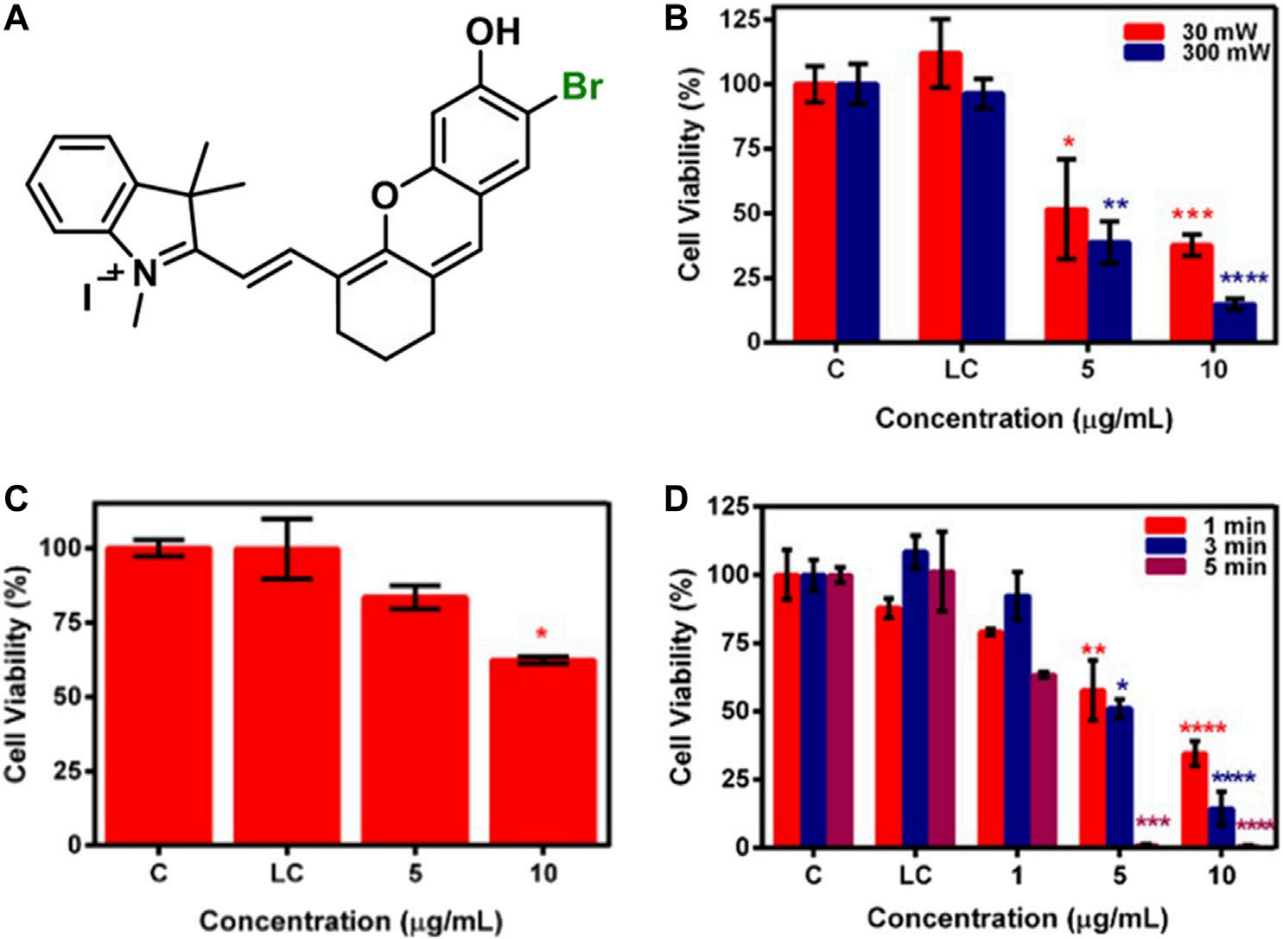
**(A)** Representative structure of brominated hemicyanine (HC-1), cell viability of brominated hemicyanine treated SW480 cell line after **(B)** 640 nm, **(C)** 808 nm, and **(D)** 640 + 808 nm laser irradiation. Reproduced from ref. Gunduz et al. (2021) with permission from Elsevier, copyright 2021.

In our previous work, we investigated PTT potential of brominated hemicyanine (Figure 12A) under a single (640 or 808 nm) and dual laser (640 + 808 nm) irradiation in addition to its PDT potential. A 7.8-, 17-, and 21.6°C temperature increase was observed after 640, 808, and 640 + 808 nm laser irradiation, respectively. The photothermal conversion efficiency was calculated as 50% for 808 nm and 57% for 640 nm. Both single 640 and 808 nm caused a significant cell death at 30 and 300 mW (Figure 12B) and 1,000 mW (Figure 12C) laser power, respectively, on HeLa and SW480 cell lines after 5-min irradiation. However, dual laser mode (640 nm/300 mW, 0.78 W/cm^2^ + 808 nm/1,000 mW, 2.6 W/cm^2^) induced a significant cell death even after 1- and 3-min irradiation and provided complete cell death on both cell lines after 5-min irradiation due to synergistic phototherapy (Figure 12D) (Gunduz et al., 2021).

Tables 1, 2 summarize the discussed phototherapy studies wherein heavy atom–modified cyanine dyes were used.

**TABLE 1:** Cell death results in phototherapy of heavy atom–modified cyanine dyes.

Sample	Type of action	Cell type	Irradiation wavelength (nm)	Irradiation time (min)	Irradiation power density	Concentration	Cell death (%)	Ref
2bb	PDT	HepG2	780	10	3.8 mW/cm^2^	10 µM	Complete	Usama et al. (2018)
IR8	PDT + PTT	HeLa	808	5	1 W/cm^2^	100 µg/ml	≈90	Liu et al. (2021)
ICy-N	PDT	4T1	660	5	12 mW/cm^2^	1.5 mM	80	Xu et al. (2019)
Iodinated derivative of IR-783	PDT	BxPC-3	780	1	100 mW	50 µM	>90	Atchison et al. (2017)
		MIA-PaCa-2					60	
PNPS	PDT + Chemo	HeLa	White light	180	50 W	30 µM	≈90	Liu et al. (2017)
		HepG2					>90	
CyI	PDT	HepG2	808	1	0.3 W/cm^2^	50 µg/ml	65	Cao et al. (2019)
	PTT + PDT	HepG2	808	1	0.96 W/cm^2^	50 µg/ml	95	Cao et al. (2019)
IR825-Cl	PTT	HeLa	808	5	1 W/cm^2^	20 µg/ml	>90	Pan et al. (2017)
HC-1	PTT + PDT	SW480	640	5	300 mW	10 µg/ml	85	Gunduz et al. (2021)
		HeLa				7.5 µg/ml	80	
	PTT + PDT	SW480	808	5	1000 mW	10 µg/ml	38	Gunduz et al. (2021)
		HeLa				7.5 µg/ml	63	
	PTT + PDT	SW480	640 + 808	5	300 + 1000 mW	10 µg/ml	Complete	Gunduz et al. (2021)
		HeLa				7.5 µg/ml	92	

**TABLE 2:** *In vivo* results in phototherapy of heavy atom–modified cyanine dyes.

Sample	Type of action	Tumor type	Irradiation wavelength (nm)	Irradiation time (min)	Irradiation power density	Injected sample	Inhibition rate (%)	Ref
Iodinated derivative of IR-783	PDT	BxPC-3	780	9	100 mW	2.5 mg/kg	≈100	Atchison et al. (2017)
ICy-N	PDT	4T1	660	20	100 mW/cm^2^	50 µM	≈100	Xu et al. (2019)
CyI	PDT	HepG2	808	1	0.3 W/cm^2^	1.5 mg/kg	100	Cao et al. (2019)
	PTT + PDT	HepG2	808	1	0.96 W/cm^2^	1.5 mg/kg	100	Cao et al. (2019)

## Conclusions and Outlook

In this mini-review, we summarized the recent studies on cyanine dye–based PDT and PTT. Although PDT and PTT offer many advantages over other techniques, the combination of the two brings about a strong synergism that makes the phototherapies applicable over a variety of tumor types. In doing so, finding a single PS that can perform both tasks, especially at a single and preferentially at NIR is critical in going forward in terms of cost and practicality. Cyanine dyes have appeared as promising *in vivo* phototherapy agents, which have demonstrated the ability to perform the combination of PTT and PDT as a single agent under laser irradiation. However, their poor singlet oxygen quantum yields limit the efficacy of the therapeutic outcome. Decoration of these dyes with heavy atoms substantially solved this issue and provided successful results at low concentrations, low irradiation powers, and short irradiation time. We believe that new-generation cyanine derivatives as effective phototherapy agents will continue to appear and more promising results will come by further improving the selectivity of these agents toward cancer cells. We also anticipate that studies that are aiming to investigate pharmacokinetics and pharmacodynamics of these agents are still needed in order to fasten the clinical translation of such designs.
